# Copper-catalyzed generation of nitrogen-centered radicals and reactions thereof

**DOI:** 10.24820/ark.5550190.p012.078

**Published:** 2023-11-03

**Authors:** Sherry R. Chemler

**Affiliations:** Chemistry Department, Natural Science Complex, State University of New York at Buffalo, Buffalo, New York 14260, United States of America

**Keywords:** Nitrogen-centered radicals, copper-catalyzed, copper

## Abstract

Nitrogen-centered radicals are reactive intermediates that can function in the formation of new C–N bonds or enable the formation of other bonds through their ability to abstract hydrogen atoms to generate carbon radical intermediates. Methods for the generation of nitrogen-centered radicals have traditionally involved application of radical initiators and propagators such as peroxides, AIBN and tin hydrides. More recently, approaches to nitrogen-centered radicals involving copper catalysis have been developed. In the transformations summarized herein, the copper catalyst either oxidizes or reduces the nitrogen-centered radical precursor. Some of these methods have been developed as catalytic enantioselective using chiral copper complexes.

## Graphical Abstract

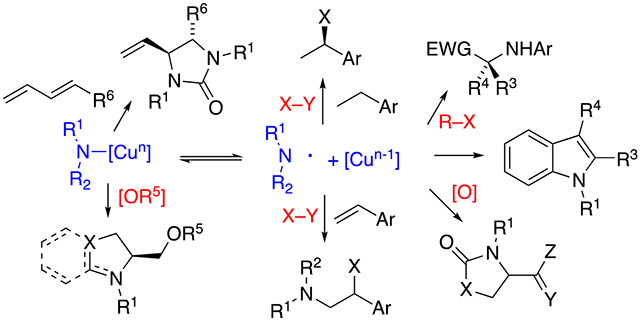

## 1. Introduction

**Scheme 1.**
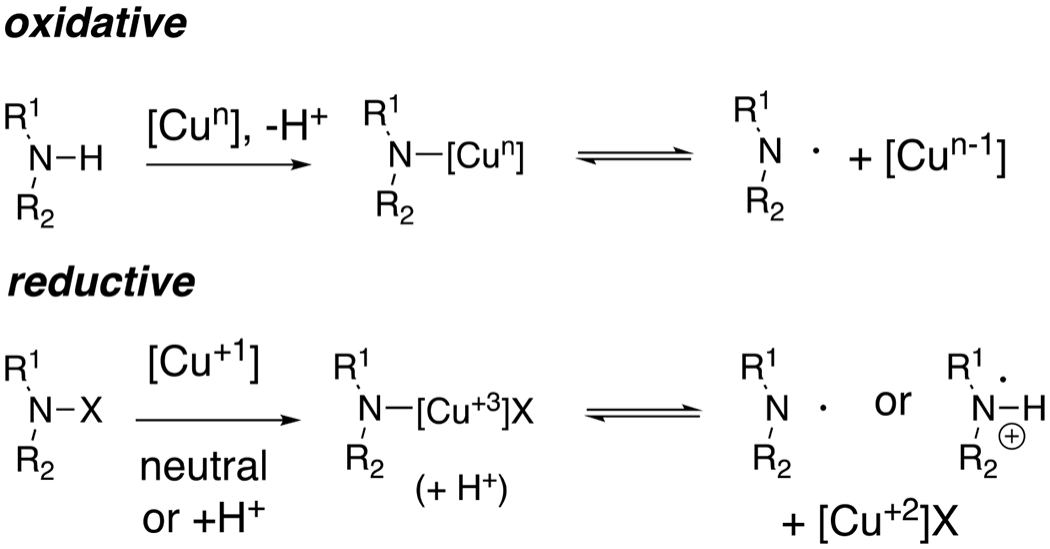
Modes of [Cu]-catalyzed nitrogen-centered radical formation.

The use of nitrogen-centered radicals in organic synthesis has been practiced and appreciated since the late 20^th^ century, and powerful applications in complex natural product synthesis have been demonstrated.^1^ Hydrogen atom transfer (HAT) by nitrogen-centered radicals has enabled subsequent C–C, C–O and C–N bond formations,^2^ and addition of nitrogen-centered radicals to unsaturated C–C bonds can result in direct C–N bond formation.^1^ The production and application of nitrogen-centered radicals via reduction of N-X bonds by applying radical initiation techniques such as AIBN/Bu_3_SnH, peroxides and thermolysis or photochemical cleavage of weak bonds has been particularly developed and reviewed by Zard.^3^ More recently, the generation of nitrogen-centered radicals has been achieved by applying visible light photocatalytic^4–6^ and electrochemical^7^ activation of amine derivatives. Nitrogen-centered radicals can also be accessed by oxidation of a precursor amine derivative or by reduction of an N-X derivative by copper salts of the appropriate oxidation state (Scheme 1).

Because of its range of oxidation states, copper has the ability to generate nitrogen-centered radicals via reduction of N-X bonds [using Cu(I)] as well as by oxidation of suitable N-H bonds [Cu(II) and Cu(III)]. The reactivity of an amine derivative toward oxidation or reduction impacts its ability to form a nitrogen-centered radical upon treatment with a copper salt. Some nitrogen radicals have been observed spectroscopically to be complexed with [Cu(I)] and [Cu(II)],^8–10^ and have been characterized as persistent radicals. Such complexation likely plays a role in many of the reactions described herein.

One advantage of the copper-catalyzed methodologies is the ability of copper salts to participate in subsequent mechanistic bond-forming steps with the radical species generated in the reaction. Notably, catalytic enantioselective transformations are often enabled when copper complexes coordinated to enantioenriched chiral ligands are applied.

This review is organized by copper’s role, either as an oxidizing or reducing agent, which in turn depends on the functionalization level of the amine derivative, e.g., R^1^R^2^N–H functionalized or R^1^R^2^N–X. It is further organized by the electronic effects of the amine derivative’s R^1^ and R^2^ substituents, which may be electron-donating or electron-withdrawing.

## 2. Oxidative Formation of Nitrogen-centered Radicals and Reactions Thereof

The direct one-electron oxidation of amines and their derivatives by copper salts can generate nitrogen-centered radicals. Not all amine derivatives will undergo this oxidation. Generally, the amine or amine derivative must be sufficiently electron-rich or its radical must be sufficiently stabilized by resonance.^11–12^

### 2.1. Reactions of anilines

**Scheme 2.**
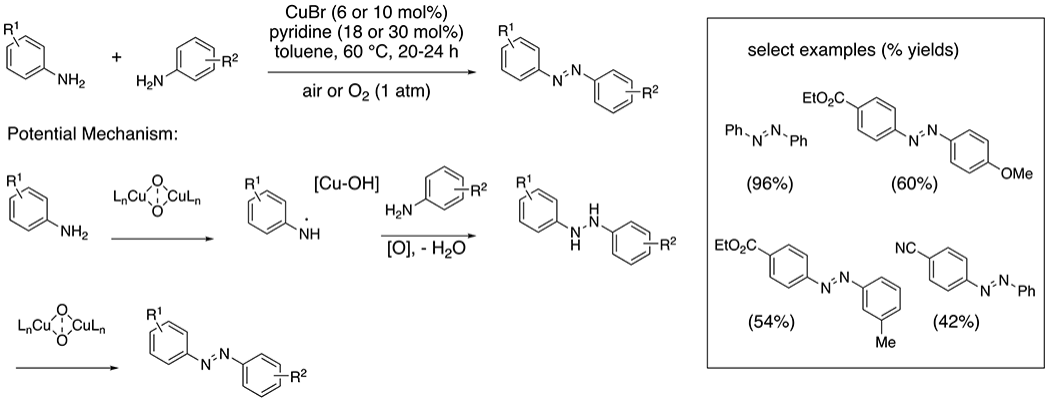
Oxidative coupling of anilines to give diazines.

Primary anilines readily undergo copper-catalyzed dehydrogenative coupling under aerobic conditions to provide aromatic azo compounds (Scheme 2).^13–14^ The aniline scope is more broad when the reaction is performed in the presence of pyridine, which may act as a ligand or base. Electron-rich anilines are more reactive than electron-poor anilines, which correlates with their oxidation potentials.^12^ Upon single electron oxidation, anilines form resonance stabilized aminyl radicals. While it is feasible the copper salt coordinates to both coupling partners as part of the N–N bond formation mechanism, the direct coupling of nitrogen-centered radicals is precedented under basic aerobic conditions in the absence of transition metal, although the reactions require higher temperature.^15^ Cross-coupling of different anilines was achieved by controlling reactant stoichiometry (using the less reactive aniline in excess). Higher catalyst loading and O_2_ concentration were used in the cross-coupling reactions.

**Scheme 3.**
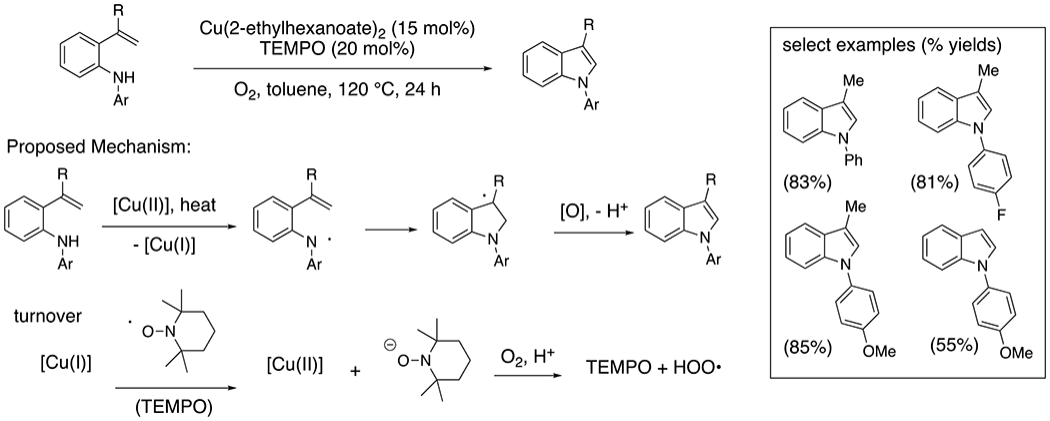
Synthesis of indoles by oxidative cyclization of 2-vinylanilines.

2-Vinyl-*N*-arylanilines undergo 5-endo-trig cyclization to provide *N*-arylindoles in the presence of copper (II) salts using either MnO_2_ or O_2_ and catalytic TEMPO as the terminal oxidant (Scheme 3).^16–17^ In the case of the aerobic reaction, catalytic TEMPO significantly impacts the efficiency of the reaction and is proposed to facilitate the re-oxidation of [Cu(I)] to [Cu(II)]. 2-Vinylanilines lacking the *N*-aryl substituent were unreactive.

### 2.2. Reactions of *N*-alkylamines

**Scheme 4.**
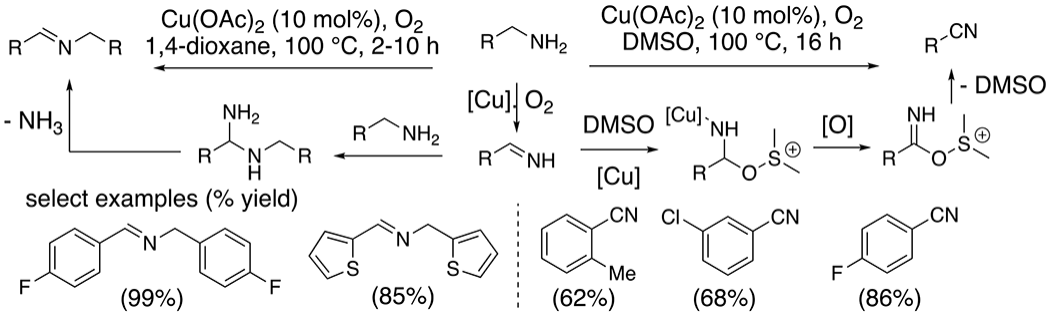
Oxidation of alkylamines to imines and nitriles.

The oxidation of amines with alpha-hydrogens, catalyzed by copper salts under oxidizing conditions, results in the formation of imines and nitriles (Scheme 4).^18^ The mechanism for these conversions is thought to involve oxidation of the amine via single electron transfer (SET) processes involving nitrogen-centered radicals.^19^ In the set of reactions illustrated in Scheme 4, the solvent determines if an imine or nitrile is formed. Initial amine oxidation can result in a common imine intermediate that can undergo addition by a second equivalent of amine, to provide the “dimeric” imine product, or, alternatively, DMSO can potentially add to the imine, and subsequent oxidation can result in the nitrile product. Interestingly, in the absence of copper catalyst, the amine oxidation reaction in DMSO provides the imine “dimer” product.^15^

**Scheme 5.**
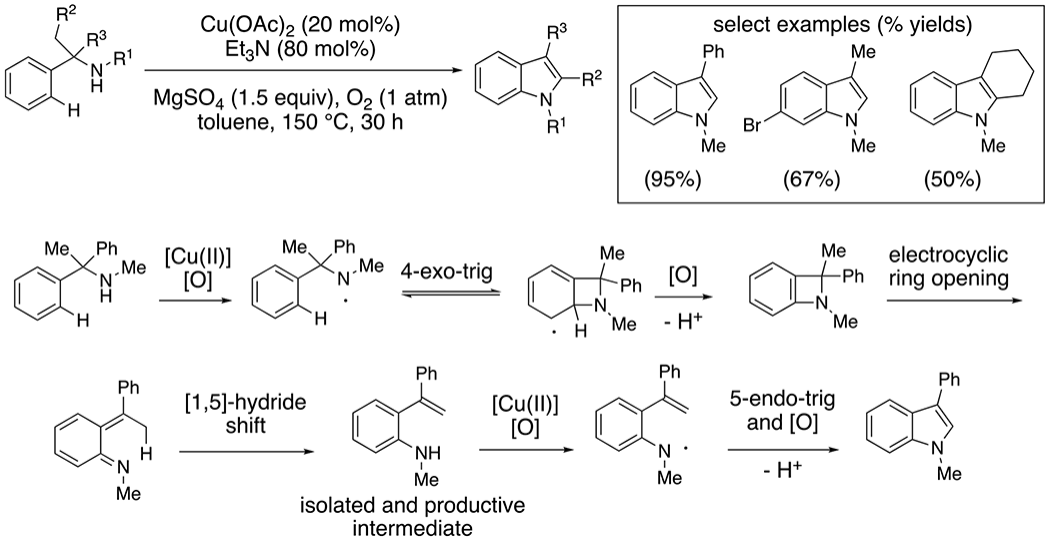
Synthesis of indoles from fully substituted benzylic amines.

When the alkyl amine lacks an alpha-hydrogen, nitrogen-centered radical reactivity can involve additions to π-bonds, as exemplified by the conversion of benzylic amines to indoles illustrated in Scheme 5, along with a proposed mechanism.^20^ Wang and co-workers proposed that the electron-rich amine is oxidized by [Cu(II)] in the presence of O_2_. They demonstrated that added base, Et_3_N, was important in the efficiency of the reaction, and may facilitate formation of an N–[Cu(II)] bond that can homolyse to the nitrogen-centered radical. Cyclization of the radical onto the arene followed by oxidation produces a strained heterobicyclic intermediate. Electrocyclic ring opening gives an exocyclic diene and 1,5-hydride shift restores aromaticity. The resulting aniline undergoes oxidation and subsequent aminyl-radical cyclization. A final oxidation provides the indole. A range of variously functionalized indoles were synthesized by this method.

### 2.3. Reactions of *N*-arylsulfonamides

**Scheme 6.**
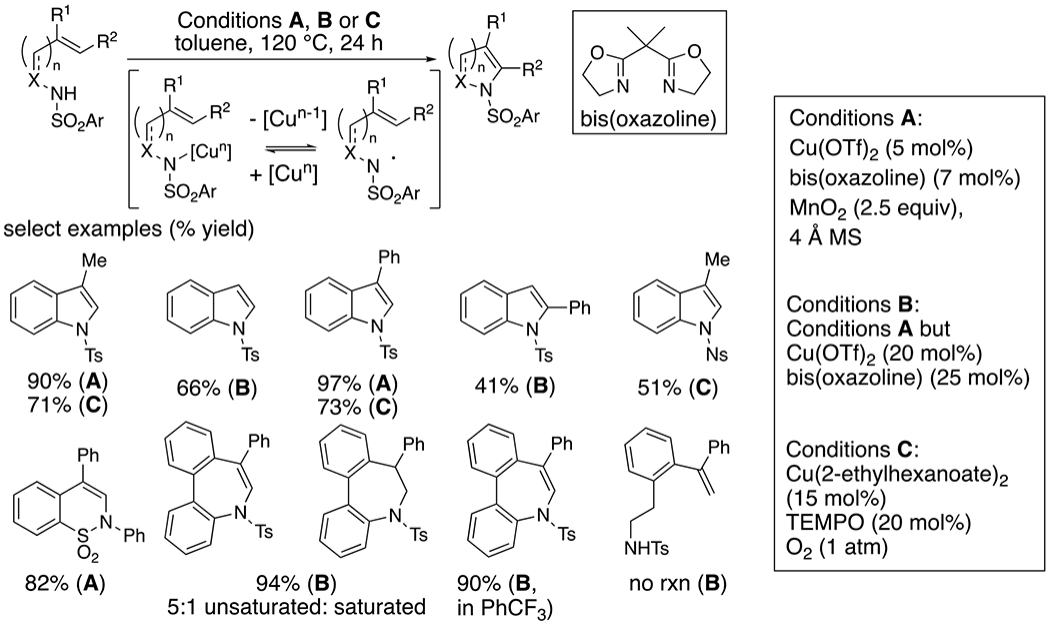
Synthesis of heterocycles from 2-vinylaryl sulfonamides.

2-Vinyl-*N*-arylsulfonamides undergo copper-catalyzed aerobic oxidative cyclization to form indoles using either MnO_2_ (conditions A and B, Scheme 6) or catalytic TEMPO with O_2_ as stoichiometric oxidant (conditions C, Scheme 6).^16–17^ While the use of O_2_ as oxidant is more environmentally benign, the scope of the reactions using MnO_2_ was broader. The scope included formation of six- and seven-membered ring heterocycles. The seven-membered ring formation provided evidence for a radical mechanism: when the reaction was run in PhCH_3_, some saturated heterocycle was obtained, which was attributed to an H-atom transfer. The unsaturated heterocycle was obtained exclusively when the same reaction was run in PhCF_3_. The requirement for an aryl ring to stabilize the nitrogen-centered radical intermediate was demonstrated by the lack of reactivity of an *N*-alkylsulfonamide (Scheme 6).

**Scheme 7.**
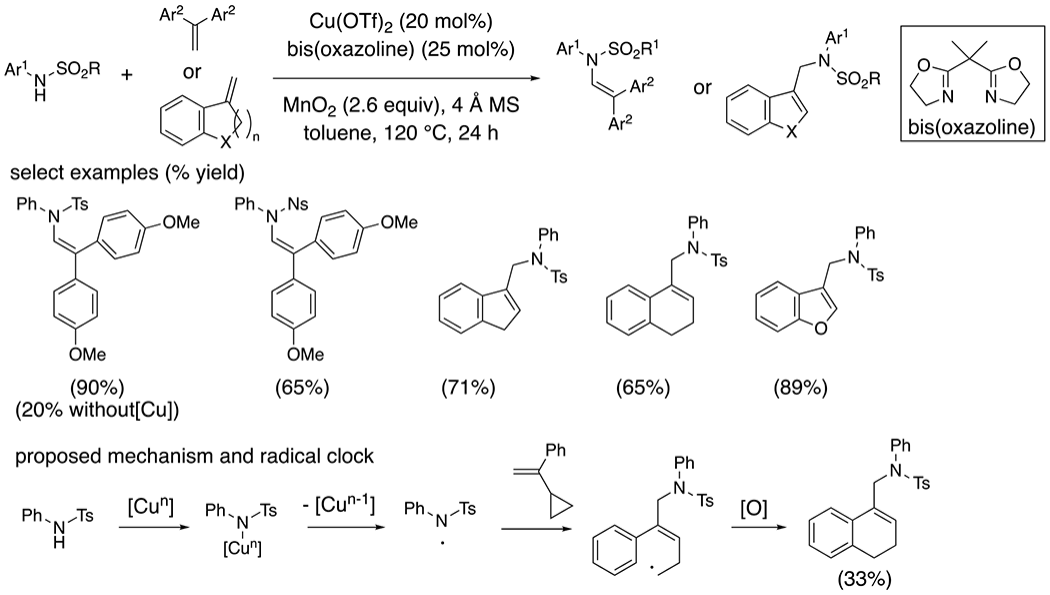
Intermolecular amination of styrenes.

Intermolecular copper-catalyzed oxidative couplings between arylsulfonamides and 1,1-diaryl ethylenes and 1-aryl-1-alkyl ethylenes were also demonstrated (Scheme 7).^16^ The resulting products are enamides or formal allylic amination adducts. The regioselectivity is consistent with a sulfamidyl radical adding to the less hindered carbon of the vinyl arene, resulting in formation of a stabilized benzylic radical. Evidence for radical reactivity was demonstrated by the radical clock substrate, 1-cyclopropyl-1-phenylethylene providing its cyclopropane ring opened adduct under the reaction conditions (Scheme 7).

**Scheme 8.**
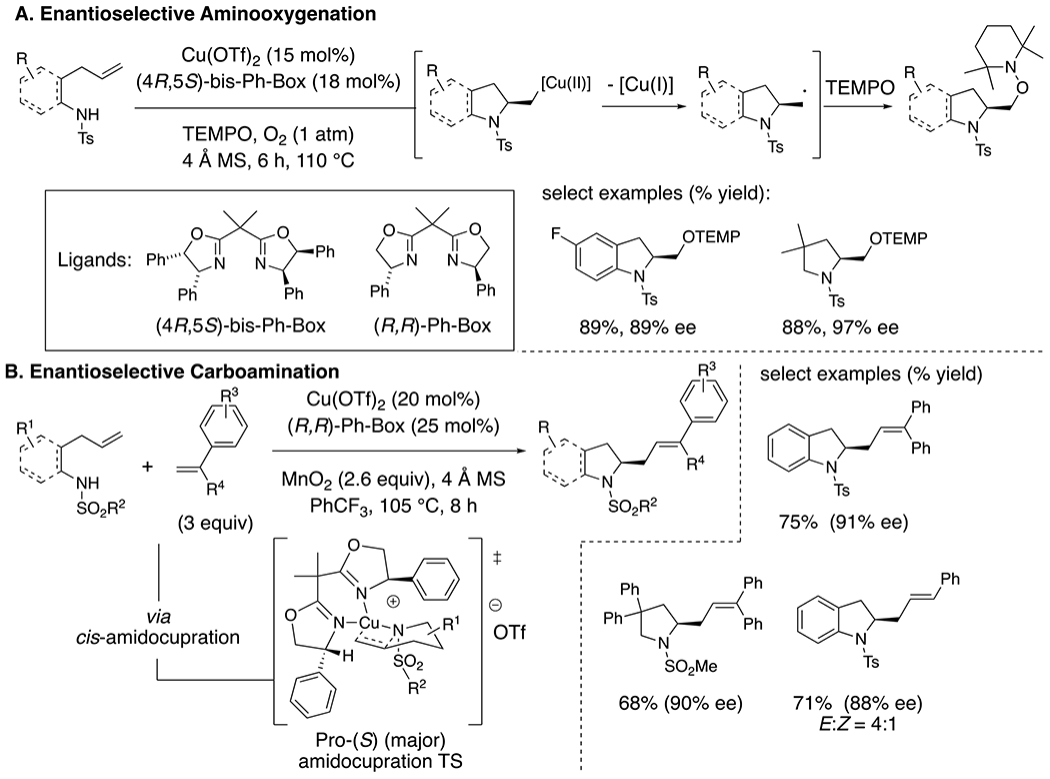
(A) Aminooxygenation and (B) carboamination of 4-pentenylsulfonamides.

If the ortho-substituent on the *N*-sulfonyl aniline is allyl instead of vinyl, 5-exo-cyclizations occur (Scheme 8).^21–25^ Similar cyclizations also occur efficiently with alkylsulfonamides. Radical reactivity in these reactions is supported by both TEMPO and diphenylethylene trapping. Application of a chiral copper catalyst, employing (*R,R*)-Ph-Box or (4*R*,5*S*)-bis-Ph-Box as ligands, results in the enantioselective synthesis of indolines and pyrrolidines (Scheme 8). Taking advantage of this polar-radical reactivity strategy, a family of related alkene difunctionalization reactions have been developed for the synthesis of enantioenriched saturated heterocycles.^25–28^

The enantioselectivity of these aminooxygenation and carboamination reactions indicates the chiral copper complex is coordinated to the substrate in the N–C bond-forming step, which is proposed to occur via a *cis*-amidocupration across the alkene (Scheme 8).^23^ An N–[Cu(II)] intermediate, formed from *N*-tosyl-2-allylaniline and Cu(2-ethylhexanoate)_2_ was observed by EPR spectroscopy and was shown to be productive in the aminooxygenation reaction with TEMPO.^29^ Formation of an alkyl radical via (a reversible)^30–31^ C–[Cu(II)] bond homolysis is proposed to occur after the enantiodetermining step.

**Scheme 9.**
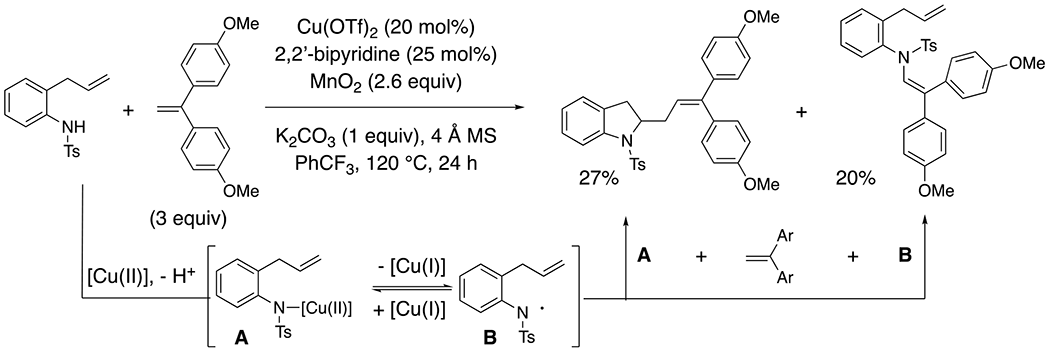
Competitive carboamination and C-H amination with an excellent radical acceptor.

Interestingly, a mixture of enamide and indoline was singularly observed with 4,4’-(ethylene-1,1-diyl)bis(methoxybenzene) (Scheme 9).^16^ This diaryl alkene’s high radical accepting ability renders it competitive with the intramolecular alkene addition reaction.

The ability of *N*-arylsulfonamides to form both enamides and indoles, as well as enantioenriched indolines has been rationalized by a reversible N–[Cu(II)] homolysis where the nitrogen-centered radical **B** is stabilized by resonance (Scheme 9). The energetics of the subsequent mechanistic step then determines if the course of the reaction proceeds via a nitrogen-centered radical pathway (e.g. alkene addition, forming a stabilized benzylic radical) or an N–[Cu] facilitated organometallic pathway (e.g. enantioselective *cis*-amidocupration alkene addition, initially forming an unstable alkyl C–[Cu(II)] bond.^30–31^ The EPR spectrum of an arylsulfonamide N–[Cu(II)] intermediate **A** indicated the presence of the N–[Cu(II)] bond, but the spectrum was dominated by the copper(II) signal compared to the nitrogen-centered radical signal, indicating most of the spin associated with the unpaired electron was associated with the copper center.^29^ Density-functional theory calculations on intermediate **A** indicated the copper center bears ca. 50% of the spin while the sulfonamide nitrogen and its aryl substituent bear ca. 30% of the spin.^32^ This indicates the arylsulfonamide has nitrogen-centered radical character, but it is not dominant. As seen in the couplings with vinylarenes (Schemes 6 and 7), the nitrogen-centered radical reactivity can dominate in the absence of a lower energy pathway, or it can compete when the vinylarene is a particularly good radical acceptor (Scheme 9). In the case of *N*-alkylsulfonamides, a nitrogen-centered radical is less readily formed due to the lower resonance stabilization of the resulting radical (and hence higher oxidation potential of the alkylsulfonamide) (Scheme 6).^25^

### 2.4. *N*-alkoxyamides and *N*-alkoxycarbamates

**Scheme 10.**
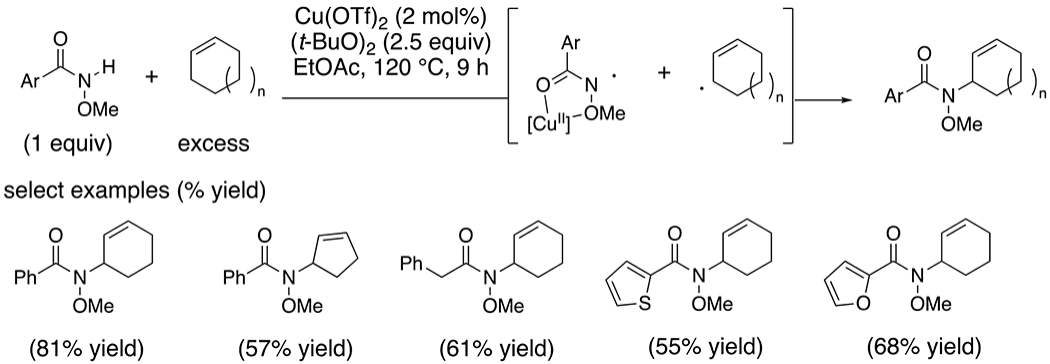
Allylic amination via copper-catalyzed coupling of *N*-alkoxy amides and alkenes.

*Intermolecular* Lei and co-workers provided EPR evidence for the involvement of a copper-complexed nitrogen-centered radical when *N*-methoxybenzamide was treated with di-*tert*-butylperoxide followed by complexation with Cu(OTf)_2_.^8^ This complex was implicated in subsequent N–C bond formations involving coupling with in situ formed allylic radicals (Scheme 10).

**Scheme 11.**
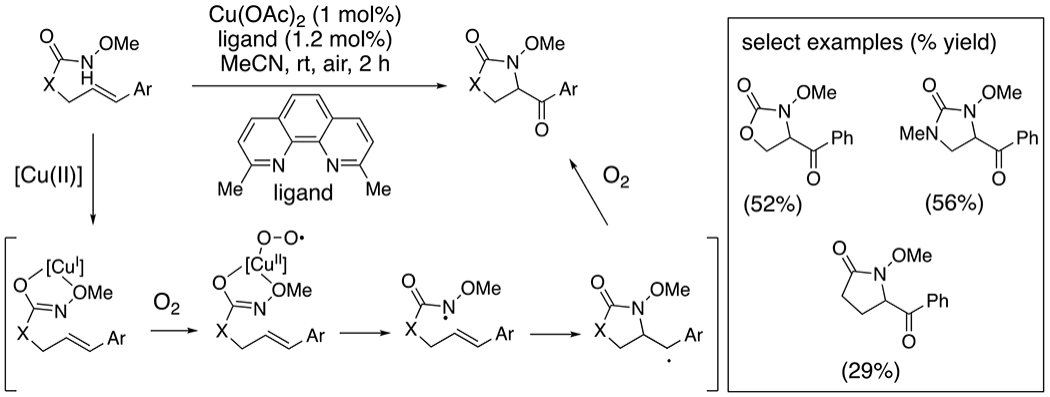
Synthesis of 5-benzoyl nitrogen heterocycles from *N*-methoxyamide derivatives.

*Intramolecular* Paradine and co-workers reported spectroscopic and reactivity evidence for the direct reduction of Cu(OAc)_2_ with a vinyl arene-tethered *N*-alkoxycarbamate, resulting in a Cu(I) amidyl complex (Scheme 11).^10^ Upon activation with molecular oxygen, an amidyl radical is proposed to be formed, as evidenced by N–C bond-forming cyclization onto the pendant internal styrene. The resulting benzylic radical then combines with O_2_ and, in the presence of the copper salts, provides a benzylic ketone product (Scheme 11). While reduction of Cu(II) to Cu(I) occurs via substrate coordination alone, alkene cyclization does not occur until O_2_ is introduced. Thus, O_2_ is proposed to react with the Cu(I) amidyl complex to form a superoxide that results in amidyl radical formation and subsequent cyclization onto the alkene. Terminal alkenes are unreactive, and chiral ligands do not engender enantioselective cyclization, leading the authors to conclude an amidyl radical and not an N–[Cu] complex is responsible for N–C bond formation.

### 2.5. Reactions of amidines

**Scheme 12.**
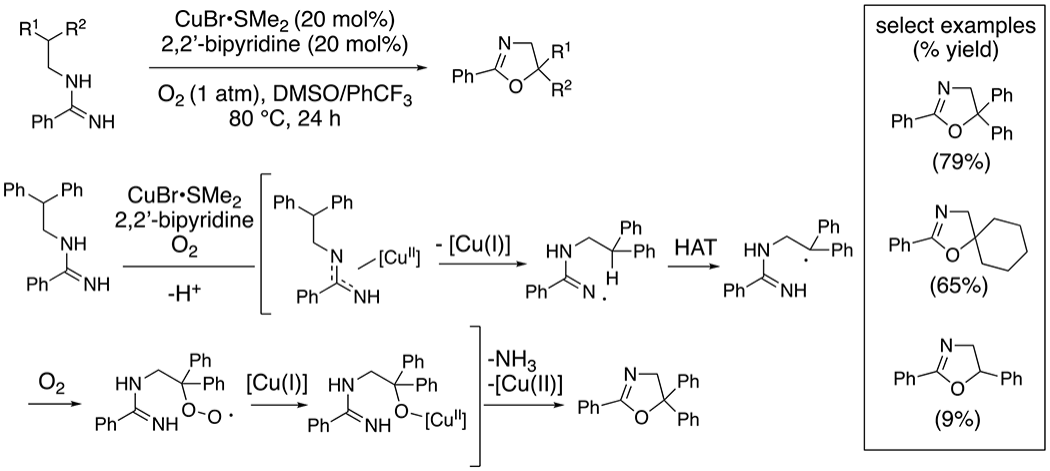
Synthesis of oxazolines from amidines.

Chiba and co-workers demonstrated copper-catalyzed oxidative C–H functionalizations of amidines bearing relatively weak pendant C–H bonds, benzylic and/or tertiary.^33^ Under aerobic conditions, amidine oxidation and cyclization provides oxazolines (Scheme 12).^34^ This reaction is thought to involve C-H abstraction by a nitrogen-centered radical intermediate, which in turn is generated by oxidation of the amidine by [Cu(II)] (Scheme 12). The resulting carbon radical forms a C–O bond with O_2_ or [Cu]–O–O•. The resulting peroxy radical is reduced to the copper alkoxide, and subsequent cyclization with concomitant extrusion of ammonia provides the oxazoline. An isotopic labelling reaction confirmed that molecular oxygen is the reaction’s oxygen source.

**Scheme 13.**
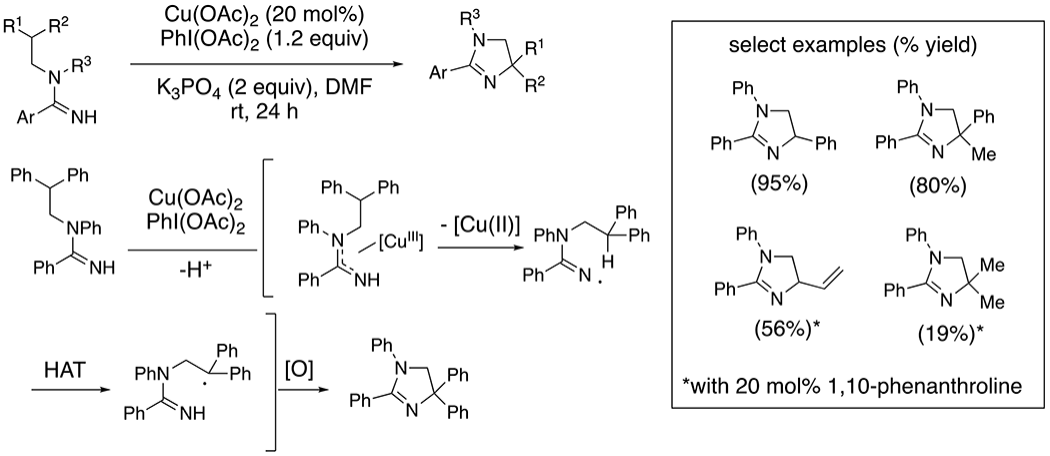
Synthesis of dihydroimidazoles from amidines.

Conversely, under anaerobic conditions using PhI(OAc)_2_ as stoichiometric oxidant, copper-catalyzed amidine oxidation and cyclization provides dihydroimidazoles (Scheme 13).^35^ This reaction takes place at lower temperature (rt vs 80 °C) and is thought to involve [Cu(III)] as the amine-oxidizing agent (Scheme 13). The resulting amidyl radical abstracts a nearby hydrogen atom (HAT) and subsequent oxidation of the carbon radical to a carbocation followed by addition of the amidine, or by reductive amination from a [Cu(III)] intermediate coordinated to both the amidine and the carbon, provides the new N–C bond (Scheme 13).

**Scheme 14.**
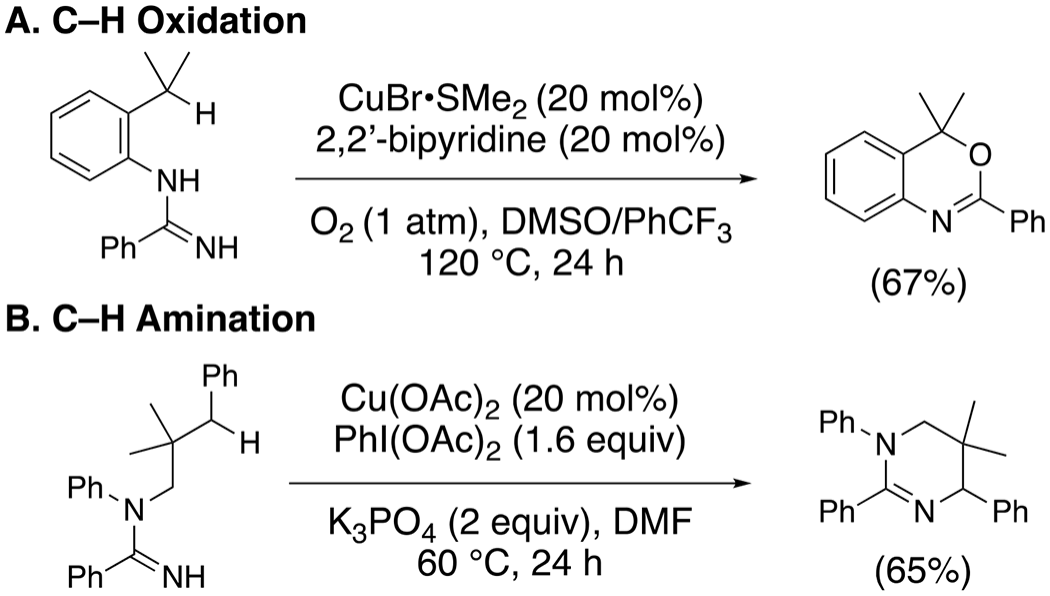
Six-membered rings from (A) C–H oxidation and (B) C–H Amination.

Six-membered rings could also be synthesized in these oxidative cyclization reactions (Scheme 14, A and B).

**Scheme 15.**
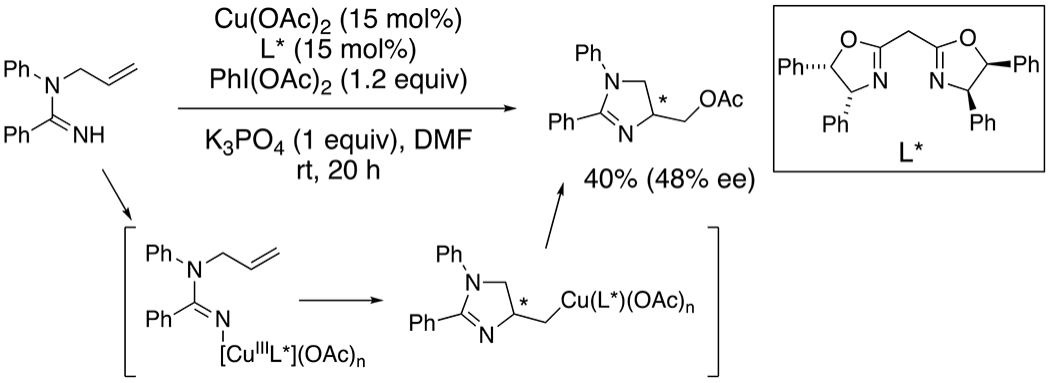
Enantioselective aminooxygenation of an *N*-allylamidine.

The reversible formation and homolysis of the N–[Cu] bond of amidines and copper ions enables both nitrogen-centered radical and organometallic N–[Cu] reactivity where the reactivity mode is determined by the functional group being acted on. An example of a N–[Cu] mechanistic mode is supported by the enantioselective aminooxygenation of a pendent terminal alkene illustrated in Scheme 15.^36^ The ligand-based asymmetric induction provides strong evidence of the chiral copper complex’s coordination to the substrate during the alkene addition step. In reactions thought to involve an amidocupration step, terminal alkenes have shown consistently higher reactivity, compared to higher substituted alkenes.

### 2.6. Reactions of *N*-aryl-carbamates and ureas

**Scheme 16.**
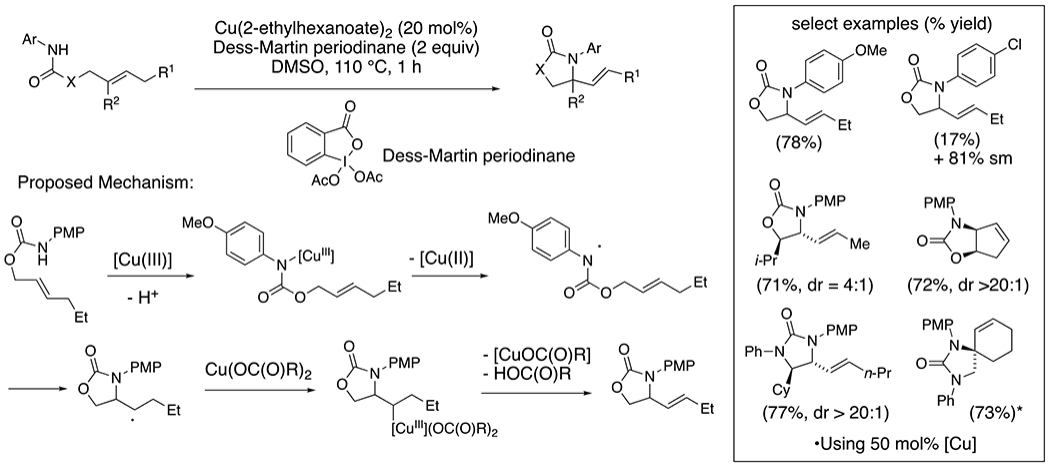
Aza-Wacker cyclization of carbamates and ureas.

Xu and co-workers have demonstrated that, in the presence of catalytic copper(II) carboxylates and the hypervalent iodine oxidant, Dess-Martin periodinane, *N*-(*p*-methoxyphenyl)carbamates and ureas tethered to internal alkenes can undergo oxidative cyclization to give 2-vinyl cyclic carbamates and ureas in a net aza-Wacker transformation (Scheme 16).^37^ The reaction is proposed to occur via initial N–[Cu(III)] bond formation followed by homolysis to give the nitrogen-centered radical. Cyclization onto the internal alkene followed by copper-facilitated elimination then provides the 2-vinyl heterocycles. The reaction does not occur either in the absence of the hypervalent iodine reagent or the copper (II) salt. Additionally, the substrate reactivity corresponds to the oxidation potential of its amide: e.g. the *p*-methoxyphenyl carbamate (*E*_P/2_ = 1.28 V vs Ag/AgCl) cyclizes in 78% yield while the corresponding *p*-chlorophenyl carbamate (*E*_P/2_ = 1.74 V vs Ag/AgCl) cyclizes in 17% yield (Scheme 16).

### 2.7. Reactions of *N*-aryl-benzamides

**Scheme 17.**
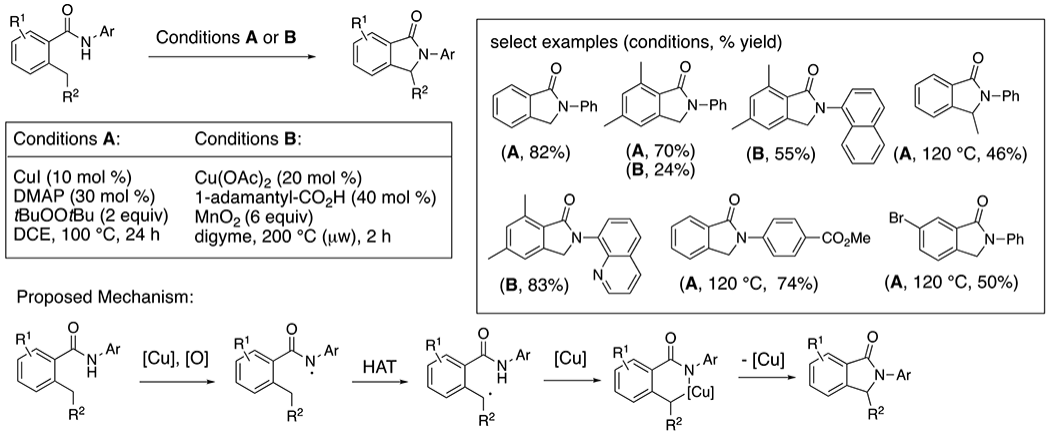
Synthesis of isoindolinones from 2-alkylbenzamides.

The synthesis of isoindolinones via copper-catalyzed intramolecular C–H amination of ortho-alkyl *N*-aryl benzamides occurs under oxidizing conditions involving either di-*tert*-butylperoxide (conditions **A**)^38^ or MnO_2_ (conditions **B**)^39^ as stoichiometric oxidant (Scheme 17). These reactions are thought to involve copper-facilitated C–N bond formation between benzylic radicals and N–[Cu]. The peroxide oxidant is stronger, enabling a more general *N*-aryl scope at lower reaction temperature.^38^ Conversely, the reaction that applies MnO_2_ as stoichiometric oxidant is highly sensitive to the *N*-aryl and benzamide substituents.^39^ While both reactions may involve formation of nitrogen-centered radicals, in the reaction employing MnO_2_, formation of a nitrogen-centered radical is more likely to occur via thermal N–[Cu] homolysis. Abstraction of the C–H bond to give the benzylic radical followed by copper-facilitated C–N bond formation then completes the isoindoline synthesis. The protocol involving the di-tert-butylperoxide is likely to involve either or both N–H and C–H abstraction by *t*-BuO· followed by copper-facilitated C–N bond formation.

### 2.8. Summary of oxidative copper-catalyzed nitrogen-centered radical formation reactions

As seen in the above examples, oxidative copper-catalyzed nitrogen-centered radical formation reactions frequently involve added base, presumably to aid in the formation of the N–[Cu^n^] bond. The nitrogen-centered radical generation is frequently thought to involve a reversible N–[Cu^n^] bond homolysis, which could be considered an inner sphere oxidation mechanism. The reactions use external stoichiometric oxidants like MnO_2_, O_2_, (*t*-BuO)_2_ and PhI(OAc)_2_. They are typically thermally activated, with reaction temperatures ranging from room temperature to 200 °C. Ease of nitrogen-centered radical generation correlates with oxidation potential of the amine derivative, where electron-rich amines tend to undergo oxidation more readily. Finally, in a number of instances the rate of reaction of the N–[Cu^n^] intermediate is greater than nitrogen-radical generation or reaction thereof, in which case organometallic mechanisms are invoked, such as ligand-influenced enantioselective alkene additions.

## 3. Reductive Generation of Nitrogen-centered Radicals and Reactions Thereof

The generation of nitrogen-centered radicals under reductive conditions often involves oxidative insertion of [Cu(I)] into an N–X bond. These reactions can take place under relatively mild reaction conditions and the mechanisms frequently involve subsequent steps that employ the copper catalyst. In many of these protocols, [Cu(I)] is formed from a [Cu(II)] pre-catalyst by in situ reduction (*vide supra*).

### 3.1. Reactions of oxime esters

**Scheme 18.**
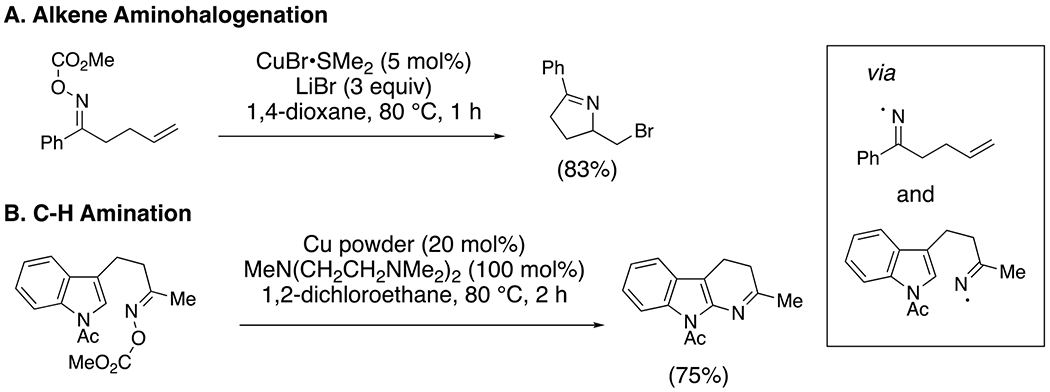
Bromoamination (A) and C-H amination (B) using oxime esters.

Zard and co-workers demonstrated a mild, single electron transfer reduction of the N–O bond of oxime esters using nickel powder as promotor in the 1990’s.^40–41^ Narasaka subsequently demonstrated that catalytic amounts of copper salts or copper powder can similarly generate iminyl radicals and achieve alkene difunctionalization^42^ and C-H animation,^43^ respectively (Scheme 18).

**Scheme 19.**
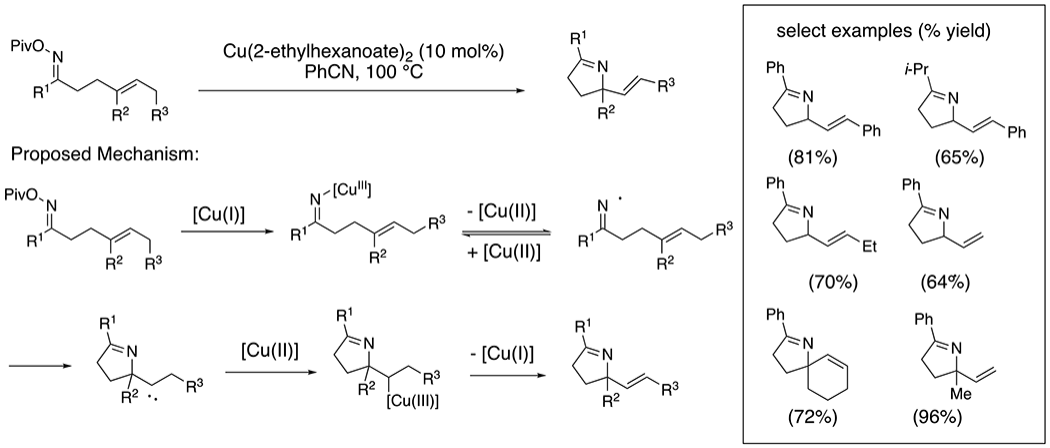
Aza-Wacker cyclization of oxime esters.

Using oxime esters as pre-activated imine derivatives, Bower and co-workers demonstrated a copper-catalyzed aza-Heck cyclization reaction for the generation of 2,5-disubstituted dihydropyrroles (Scheme 19).^44^ A mechanism involving N–O insertion by [Cu(I)], subsequent N–[Cu(III)] homolysis, addition of the resulting iminyl radical to a pendant alkene and [Cu(II)]-facilitated oxidative elimination was proposed. Evidence for the presence of an iminyl radical involving radical fragmentations and radical clock experiments was presented.

**Scheme 20.**
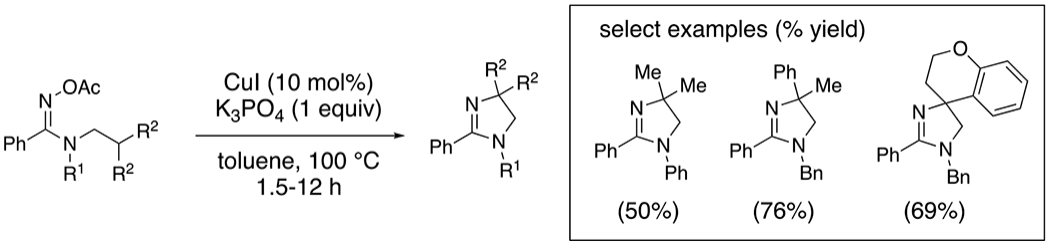
C-H amination using oxime esters.

Additionally, the C-H amination of aliphatic carbons using aminyl radicals generated by copper-activation of oximes was demonstrated by Chiba and Chen for the synthesis of dihydroimidazoles (Scheme 20).^45^ This redox-neutral transformation is a complement to the oxidative transformation developed by Chiba illustrated in Schemes 13 and 14 (*vide supra*).

### 3.2. Reactions of *O*-acylhydroxylamines

**Scheme 21.**
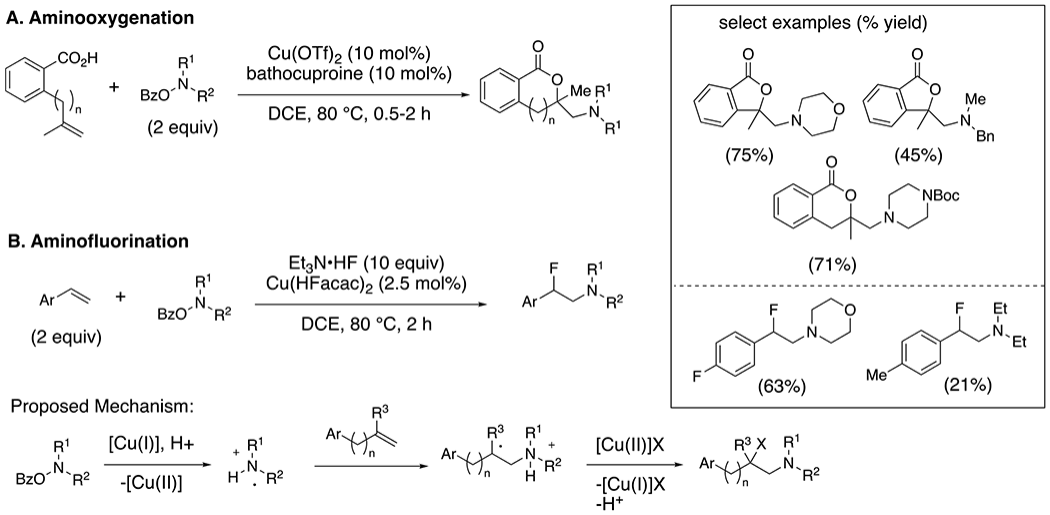
The intermolecular aminooxygenation (A) and aminofluorination (B) of alkenes.

Wang et. al have reported the application of *O*-acylhydroxylamines in copper-catalyzed nitrogen-centered radical reactions.^46–49^ For example, the intermolecular alkene addition of aminyl radicals generated from copper-catalyzed reduction of *O*-acylhydroxylamines (Scheme 21) can result in net aminooxygenations (Scheme 21A)^46–48^ and aminofluorinations^49^ (Scheme 21B). Alkene substrates that are able to form more substituted or resonance stabilized carbon radical intermediates upon addition of the aminyl radical tend to be most reactive.

### 3.3. Reactions of *N*-fluorosulfonamides

**Scheme 22.**
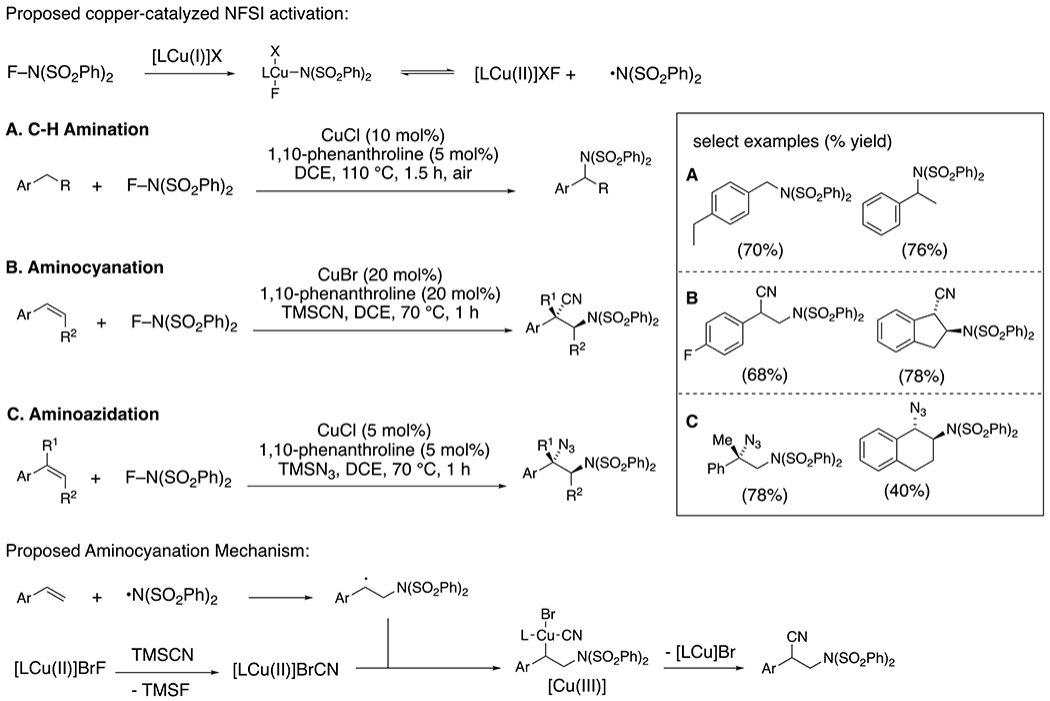
NFSI applied to C–H amination (A) and alkene aminocyanation (B) and aminoazidation (C).

Zhang and co-workers identified *N*-fluorobenzenesulfonimide (NFSI) as a reagent that can be activated by copper salts to form nitrogen-centered radicals that in turn can abstract benzylic hydrogen atoms or add to styrenes.^50^ This copper-catalyzed strategy has enabled benzylic amination^51^ as well as alkene difunctionalizations in the presence of reagents such as TMSCN^52^ and TMSN_3_.^53^ Oxidative addition of [Cu(I)] into the N–F bond of NFSI is invoked to initiate the reaction (Scheme 22). The benzylic C–H abstraction in the C–H amination process has been attributed to the bis(benzenesulfonyl)amide radical that is formed upon dissociation from the proposed [Cu(III)] catalyst intermediate (Scheme 22).^54^ In the cyanation and azidation reactions, the trimethyl silyl group of the reagents is helpful in sequestering fluoride to facilitate ligand exchange at [Cu(III)]. In these examples, addition of carbon radical intermediates to ligand-associated [Cu(II)] salts is proposed to form transient alkyl [Cu(III)] intermediates that undergo reductive elimination to form the observed benzylic-functionalized products.

**Scheme 23.**
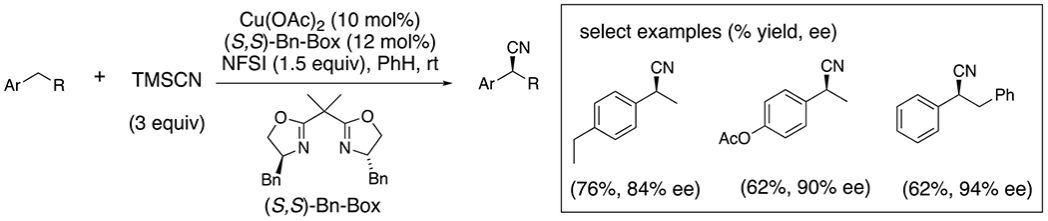
Enantioselective C–H cyanation.

Stahl and Liu subsequently developed a copper-catalyzed enantioselective benzylic C–H cyanation employing NFSI as the C–H abstracting nitrogen-centered radical reagent and TMSCN to provide the cyano group (Scheme 23).^54^ This seminal paper set the precedent for many subsequent catalytic enantioselective copper-catalyzed reactions involving benzylic radical coupling with various reagents.^55^ Of the transferable ligands on [Cu], the cyano group tends to transfer relatively rapidly and with excellent stereocontrol.

**Scheme 24.**
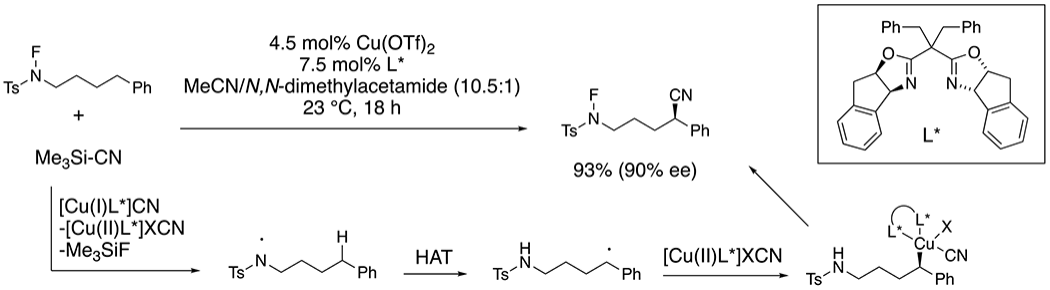
Remote enantioselective C–H cyanation.

Nagib and co-workers developed a remote enantioselective benzylic C-H cyanation involving copper-catalyzed nitrogen radical formation, intramolecular 1,5-hydrogen atom transfer and copper-facilitated CN-bond formation (Scheme 24).^56^ These amino nitriles were applied to the synthesis of enantioenriched piperidines (not shown).

### 3.4. Reactions of diaziridinones

**Scheme 25.**
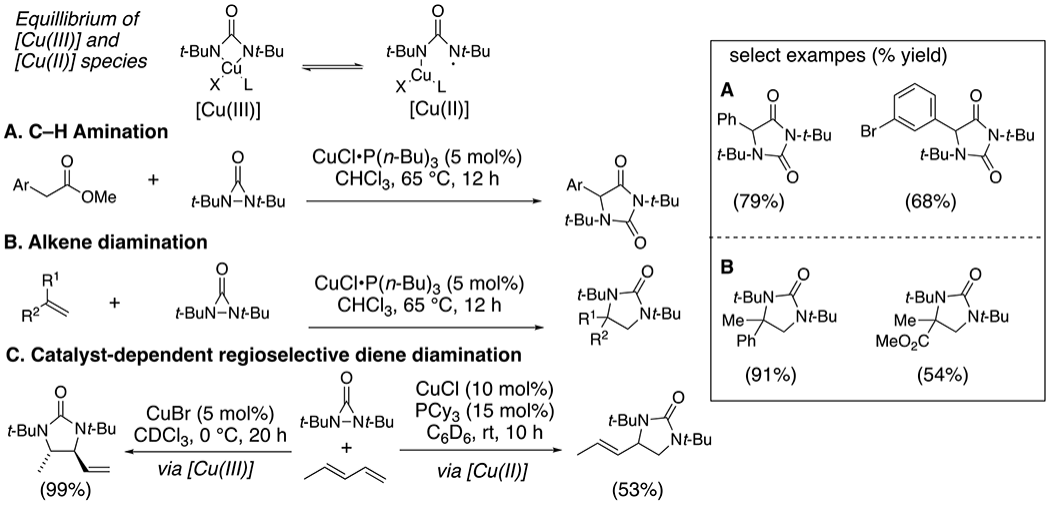
C–H amination (A), alkene deamination (B) and regioselective diene deamination (C).

Shi and co-workers have developed a family of copper-catalyzed C–H amination and alkene diamination reactions by applying diaziridinones as pre-activated nitrogen-centered radical precursors (Scheme 25).^57^ They applied copper(I) salts complexed to phosphine ligands to activate the N–N bond of diaziridinones via oxidative addition. The resulting copper complexes can be drawn as [Cu(III)] complexes in equilibrium with their [Cu(II)]-amidyl radical resonance structure. In the reactions that involve added phosphine ligand, the C–H abstraction^58^ and alkene addition^59–60^ is attributed to the amidyl radical of the [Cu(II)] complex (Scheme 25). The diamination of the internal alkene of dienes is attributed to the preferred electrophilic reactivity of the [Cu(III)] complex, which is thought to predominate in the absence of the phosphine ligand.^60^

### 3.5. Coupling of anilines with tertiary R-X

**Scheme 26.**
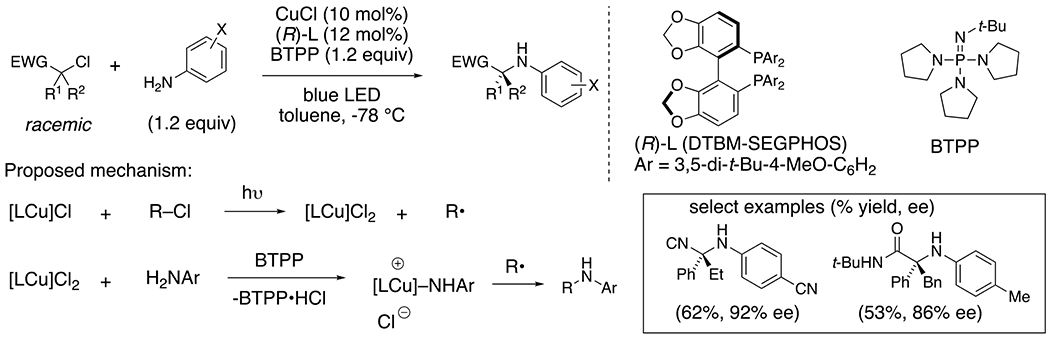
Synthesis of enantioenriched alkyl amines from racemic alkyl halides.

Fu, Peters and co-workers have developed an enantioselective, de-racemizing photocatalyzed coupling of tertiary, racemic α-chloro and α-bromonitriles or amides with anilines (Scheme 26).^61^ The mechanism is thought to involve the coupling of an in situ generated alkyl radical with a copper-aniline complex that, based on molecular modelling calculations, has significant aminyl radical character, represented as either [LCu(II)]–NHAr or [LCu(I)]·NHAr. The reaction is initiated by reduction of the alkyl halide with the photoactivated [Cu(I)] complex, generating the alkyl radical. Coordination of the aniline to the resulting [Cu(II)] and coupling with the alkyl radical via enantioselective reductive elimination (possibly via a [Cu(III)] intermediate) provides the C–N bond enantioselectively.

### 3.6. Summary of reductive copper-catalyzed nitrogen-centered radical reactions

The nitrogen-centered radical formations in these reductive copper-catalyzed reactions tend to occur via homolysis of N–[Cu(III)] bonds, and are represented as in equilibrium with nitrogen-centered radicals and [Cu(II))] salts. An exception is the photochemical coupling of anilines with tertiary halides, section 3.5, which involves radical character of amines coordinated to Cu(II) salts, as is more characteristic of the oxidative reactions in section 2.0.^61^ Subsequent [Cu(III)] intermediates generated in these processes can undergo reductive elimination, frequently with enantiocontrol. These reactions occur between a temperature range of −78 to 100 °C, where the formation of the nitrogen-centered radical is likely rate-limiting. In these reactions, at least one of the substrates is pre-oxidized, possessing an N–X or R–X bonds, so no additional oxidants are required. Finally, it is noted that the nitrogens involved in these reactions tend to be functionalized with electron-withdrawing groups, e.g. -SO_2_R, or they become protonated under the reaction conditions, rendering the nitrogen-centered radicals electron-deficient.

## 4. Conclusions

Remarkable progress has been made over the past twenty years in the area of copper-catalyzed nitrogen-centered radical generation, and the development of reactions thereof. Given the importance of C–H functionalization and C–N bond forming reactions in organic synthesis, these methods show promising broad utility. Additional significant methodological advances are anticipated in this very active research field, some potentially involving photocatalysis, which enables lower reaction temperatures.^6,61–62^ Since copper is an earth abundant transition metal and has excellent ability to generate and tame reactive radical species, it is feasible that these and related methods may be adapted for use in the chemical industry.^63^
